# Author Correction: Aerosol and surface contamination of SARS-CoV-2 observed in quarantine and isolation care

**DOI:** 10.1038/s41598-020-70939-6

**Published:** 2020-08-12

**Authors:** Joshua L. Santarpia, Danielle N. Rivera, Vicki L. Herrera, M. Jane Morwitzer, Hannah M. Creager, George W. Santarpia, Kevin K. Crown, David M. Brett-Major, Elizabeth R. Schnaubelt, M. Jana Broadhurst, James V. Lawler, St. Patrick Reid, John J. Lowe

**Affiliations:** 1 University of Nebraska Medical Center, Omaha, NE USA; 2 National Strategic Research Institute, Omaha, NE USA; 3 United States Air Force School of Aerospace Medicine, San Antonio, TX USA

Correction to: *Scientific Reports* 10.1038/s41598-020-69286-3, published online 29 July 2020

This Article contains an error.

As a result of an error during figure assembly, NQU A Windowsill Day 5 image in Fig 2B duplicates NQU Hallway Air Day 8 image in Fig 2A. The correct image for NQU A Windowsill Day 5 is shown below as Figure 1.

**Figure 1:**
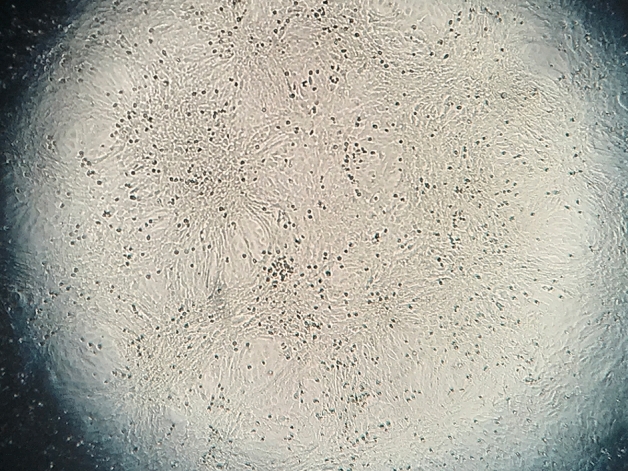
Correct image for NQU A Windowsill Day 5 cell culture.

This change does not affect the conclusions of the Article.

