# Supplementary material for: Aerosol and surface contamination of SARS-CoV-2 observed in quarantine and isolation care
Source: Sci Rep. 2020 Jul 29;10:12732. doi: 10.1038/s41598-020-69286-3 (PMC7391640; doi:10.1038/s41598-020-69286-3)
Supplement: Supplementary file 1 — Supplementary information. [file 41598_2020_69286_MOESM1_ESM.pdf]

Aerosol and Surface Contamination of SARS-CoV-2 Observed in Quarantine and Isolation Care

Authors: Joshua L. Santarpia[1,2*], Danielle N. Rivera[2], Vicki L. Herrera[1], M. Jane Morwitzer[1], Hannah M. Creager [1], George W. Santarpia[1], Kevin K. Crown[2], David M. Brett-Major[1], Elizabeth R. Schnaubelt[1,3], M. Jana Broadhurst[1], James V. Lawler[1,2], St. Patrick Reid[1], and John J. Lowe[1,2]

Affiliations:

[1]University of Nebraska Medical Center

[2]National Strategic Research Institute

[3]United Stated Air Force School of Aerospace Medicine

Supplementary Information

Room	Day	Bedside Table or Bed Rail (copies/μL)		Air Handling Grate (copies/μL)		Floor Under Bed (copies/μL)		Room Window Ledge (copies/μL)		Misc. Personal Items (copies/μL)			Phones (copies/μL)		Remote (copies/μL)		Toilet (copies/μL)		Air Samples (copies/L of air)		Samples Collected	% Positive	Oral Temp. (˚C)	Other Sym?
		Average	Stand. Dev	Average	Stand. Dev	Average	Stand. Dev	Average	Stand. Dev	Description	Average	Stand. Dev	Average	Stand. Dev	Average	Stand. Dev	Average	Stand. Dev	Average	Stand. Dev				
NQU A	5	1.31	0.11					0.65	0.29				0.14	0.24	0.12	0.21	0.95	0.39	4.00	6.93	6	100.0%	39.1	Y
NQU B	5	UND	NC					0.23	0.40				0.35	0.33	UND	NC	UND	NC	2.79	4.84	6	50.0%	36.8	Y
NQU C	5	UND	NC					0.25	0.43				0.15	0.25	0.22	0.20	0.28	0.48	8.34	7.25	6	83.3%	37.3	N
NQU E	6	0.12	0.20					0.21	0.36				UND	NC	0.41	0.12	0.11	0.20	2.70	4.68	6	83.3%	38.1	Y
NQU F	6	0.31	0.27					UND	NC				0.41	0.21	UND	NC	0.53	0.24			5	60.0%	37.0	Y
NQU G	6	UND	NC					UND	NC				0.32	0.30	UND	NC	0.41	0.10			5	40.0%	37.3	N
NQU H	7	0.16	0.27					0.20	0.35				UND	NC	0.28	0.25	0.39	0.38	3.30	5.72	6	83.3%	36.9	Y
NQU I	7	0.20	0.35					0.13	0.22				UND	NC			0.25	0.21	8.22	8.27	5	80.0%	37.3	N
NQU A	8	0.20	0.34					0.39	0.41	Exercise Bike	UND	NC	0.46	0.40	0.44	0.41	UND	NC	4.74	4.13	7	71.4%	38.0	Y
NQU B	8	0.31	0.33					0.25	0.23	Hot Pot	0.18	0.30	0.13	0.23	UND	NC	0.29	0.25	UND	NC	7	71.4%	36.6	N
NQU C	8	0.08	0.14					UND	NC	Hot Pot	0.42	0.36	0.10	0.17	UND	NC	0.11	0.18	5.68	9.85	7	71.4%	37.5	N
NQU D	9	0.19	0.33					0.17	0.30	iPad	0.10	0.17	0.15	0.25	UND	NC	0.55	0.18	UND	NC	7	71.4%	ND	ND
NQU E	9	UND	NC					UND	NC				0.28	0.32	0.25	0.22	0.22	0.20	UND	NC	6	50.0%	37.2	Y
NQU F	9	UND	NC					0.15	0.26				0.12	0.20	UND	NC	0.09	0.16	UND	NC	6	50.0%	37.1	Y
NQU G	9	0.46	0.40					UND	NC				0.19	0.33	0.18	0.31	0.14	0.25	UND	NC	6	66.7%	37.3	N
NQU H	9	0.58	0.24					0.67	0.32	Dumb bells<br>Treadmill<br>Yoga Mat	UND<br>0.20<br>0.16	NC<br>0.34<br>0.27	UND	NC	UND	NC	0.43	0.12	4.22	7.31	9	66.7%	37.1	N
NQU I	9	0.09	0.16					0.14	0.24	iPad	0.43	0.42	0.14	0.24	0.17	0.29	0.20	0.35	UND	NC	7	85.7%	36.9	N
NBU A Patient 1	10	UND<br>0.23<br>0.11	NC<br>0.21<br>0.19	UND<br>0.20	NC<br>0.34	0.39<br>0.21	0.38<br>0.37	0.14<br>UND	0.24<br>NC	Spirometer<br>Spriometer<br>O2 Cannula	0.34<br>UND<br>0.15	0.38<br>NC<br>0.26			1.70	0.24	UND<br>0.13	NC<br>0.22	> 6ft from patient<br>2.42	 4.20	16	68.8%	36.8	Y
NBU B Patient 2	10	0.33<br>0.14<br>UND	0.31<br>0.25<br>NC	0.44<br>1.75	0.44<br>0.33	0.41<br>1.02	0.46<br>0.32	0.36<br>0.34	0.33<br>0.31	Laptop<br>Glasses<br>Pulse Ox Finger Monitor	0.19<br>0.22<br>0.56	0.18<br>0.38<br>0.48	0.13	0.23			0.21	0.37	>6f from patient<br>UND	 NC	15	86.7%	37.3	Y
NBU B Patient 3	18	1.32	0.24	1.71	0.08	0.20	0.34	0.48	0.44	Lotion tube	0.32	0.55			0.23	0.20	UND	NC	Near Patient<br>4.07<br>> 6ft from patient<br>2.48	 7.06<br><br>4.30	9	88.9%	37.3	Y
Percent Positive		70.8%		80.0%		100.0%		72.7%		75.0%			77.8%		55.6%		81.0%		63.2%				57.9%	57.9%
ρ to Fever		0.15						0.25		-0.07			-0.02		0.23		0.02		0.36		0.39			

Table S1. Results of all in-room samples collected in this study. UND denotes undetected, NC denotes Not Calculated. ND denotes No Data. The dotted background indicates the sample from which cell culture data is shown in Figure 2. In the case where no data was obtained, the patient had been transferred from another facility less than 24 hours prior to sampling.

Location	Day	Hallway Air Samples (copies/L of air)		Personal Air Samples (copies/L of air)	
NQU	5	UND	NC		
	5	UND	NC		
	6	5.76	5.10		
	6	6.00	5.90		
	7	2.08	3.60		
	7	UND	NC		
	8	8.69	3.69		
	8	2.36	4.09		
	8	2.29	3.97		
	9			7.39	19.20
				5.37	7.15
NBU	10	UND	NC		
	10	2.99	5.19		
	10	UND	NC		
	18			19.17	49.82
				48.22	67.16
Percent Positive		58.3%		100.0%	

Table S2. Results of hallway air samples and personal air samples. UND denotes undetected, NC denotes Not Calculated. The dotted background indicates the sample from which cell culture data is shown in Figure 2.
